# Enhanced Productivity of a Lutein-Enriched Novel Acidophile Microalga Grown on Urea

**DOI:** 10.3390/md9010029

**Published:** 2010-12-24

**Authors:** Carlos Casal, Maria Cuaresma, Jose Maria Vega, Carlos Vilchez

**Affiliations:** 1 CIDERTA, University of Huelva, Park Huelva Empresarial, 21007, Huelva, Spain; E-Mail: carlos.bejarano@dqcm.uhu.es (C.C.); 2 International Centre for Environmental Research (CIECEM), University of Huelva, Parque Dunar s/n, Matalascañas, Almonte, 21760, Huelva, Spain; E-Mail: maria.cuaresma@dqcm.uhu.es (M.C.); 3 Faculty of Chemistry, Department of Plant Biochemistry and Molecular Biology, University of Seville, 41012, Seville, Spain; E-Mail: jmvega@us.es (J.M.V.)

**Keywords:** urea, *Coccomyxa*, extremophile microorganisms, lutein, microalgae

## Abstract

*Coccomyxa acidophila* is an extremophile eukaryotic microalga isolated from the Tinto River mining area in Huelva, Spain. *Coccomyxa acidophila* accumulates relevant amounts of β-carotene and lutein, well-known carotenoids with many biotechnological applications, especially in food and health-related industries. The acidic culture medium (pH < 2.5) that prevents outdoor cultivation from non-desired microorganism growth is one of the main advantages of acidophile microalgae production. Conversely, acidophile microalgae growth rates are usually very low compared to common microalgae growth rates. In this work, we show that mixotrophic cultivation on urea efficiently enhances growth and productivity of an acidophile microalga up to typical values for common microalgae, therefore approaching acidophile algal production towards suitable conditions for feasible outdoor production. Algal productivity and potential for carotenoid accumulation were analyzed as a function of the nitrogen source supplied. Several nitrogen conditions were assayed: nitrogen starvation, nitrate and/or nitrite, ammonia and urea. Among them, urea clearly led to the best cell growth (~4 × 10^8^ cells/mL at the end of log phase). Ammonium led to the maximum chlorophyll and carotenoid content per volume unit (220 μg·mL^·1^ and 35 μg·mL^·1^, respectively). Interestingly, no significant differences in growth rates were found in cultures grown on urea as C and N source, with respect to those cultures grown on nitrate and CO_2_ as nitrogen and carbon sources (control cultures). Lutein accumulated up to 3.55 mg·g^·1^ in the mixotrophic cultures grown on urea. In addition, algal growth in a shaded culture revealed the first evidence for an active xanthophylls cycle operative in acidophile microalgae.

## 1. Introduction

*Coccomyxa acidophila* is a novel microalgal specie isolated from Tinto River (Huelva, Spain), which is so-called the “Red river” due to its high iron water concentration. This special feature causes the river bed to constitute an acidic environment where the pH value remains constantly between 2 and 3 along a stretch of 80 km [1]. Besides, this microalga is characterized by having important potential to accumulate high lutein concentrations, a carotenoid with powerful well-known antioxidant properties [2,3].

Nowadays, extremophile organisms are gaining increasing interest due to their faculty to be used as renewable source of different high value compounds including carotenoids, fatty acids (PUFAs), lipids, vitamins, toxins, enzymes, *etc.* [4–6]. Furthermore, the extremophile character of these microorganisms can be a benefit for getting axenic cultures with no interference from others microalgae. In general, apart from contamination risks, one of the main problems for microalgae cultivation is the relatively high costs, which is expected to be overcome by technological advances [7]. For that reason, since some time ago, efforts are being focused on reducing the cost of elements related to microalgae cultivation. One aspect that puts up the total price of the operation of production systems is the high CO_2_ demand that photosynthetic microorganisms usually have. In any case, although there are currently various attempts for capturing carbon dioxide by means of algae cultures from industrial flue gases [8], one strategy aimed to reduce costs could be the replacement of the carbon source by another cheaper option.

A wide variety of nitrogen sources, such as ammonia, nitrate, nitrite and urea, can be used as nitrogen source for growing microalgae [9]. Urea (CO(NH_2_)_2_) is a small-molecular weight polar and relatively lipid-insoluble compound which is ubiquitous in nature. This organic compound can be considered as a combined source of nitrogen and carbon and it has diverse functions. In organisms containing the enzyme urease, a nickel-dependent metalloenzyme [10] present in bacteria, fungi and plants, urea is primarily used as a source of nitrogen necessary for growth. However, since urease metabolizes urea to CO_2_ and ammonia, thus providing a ready source of base, metabolism of urea by urease can also enable microorganisms to respond to acid challenges [11]. On the other hand, in mammals, urea is the primary waste product of amino acid catabolism [12]. Urea is a versatile substance and its role largely depends on whether it is an end-product or can be further broken-down, and if so, the utilization of the break-down products also varies considerably, either for anabolic processes or for buffering under acidic conditions.

Previous works performed in our group with acidophile microalgae growing under mixotrophic conditions showed that urea can be a more than suitable alternative for cultivation of this microalga, showing good productivity and lutein accumulation results. Moreover, in the literature, several examples can be found where urea is shown to be an effective combined source of N and C for the production of *S. Platensis*, *Neochloris oleoabundans* and *Chlorella sp*. under different cultivation modes [7,9,13–16].

This work aimed at assessing the effect of different nitrogen sources on biomass productivity and carotenoid accumulation of *Coccomyxa acidophila*, paying special attention to the amount of accumulated lutein. In addition, the results will allow for assessing the use of nitrogen sources other than the conventional ones in growing acidophile microalgae.

## 2. Results and Discussion

### 2.1. Coccomyxa acidophila enhanced growth on urea

It was mentioned above that acidophile microalgae have so far never been used for massive production. Massive production requires fast growing microalgal strains. Most acidophile microalgae are slow growth strains, as reported in the literature [5,17]. However, the growth of acidophile microorganisms in acidic culture media becomes advantageous for biomass production, as under such conditions the growth of other microorganisms becomes difficult. Therefore, we attempted to find culture conditions under which *Coccomyxa acidophila* cultivation is enhanced, such that growth rates and productivity values approached those of common microalgae. In such a situation, the acidophile microalga should show fast-growth and could hopefully be grown in outdoor systems with limited risks for microbial contamination, in comparison to common microalgae.

**Figure 1:**
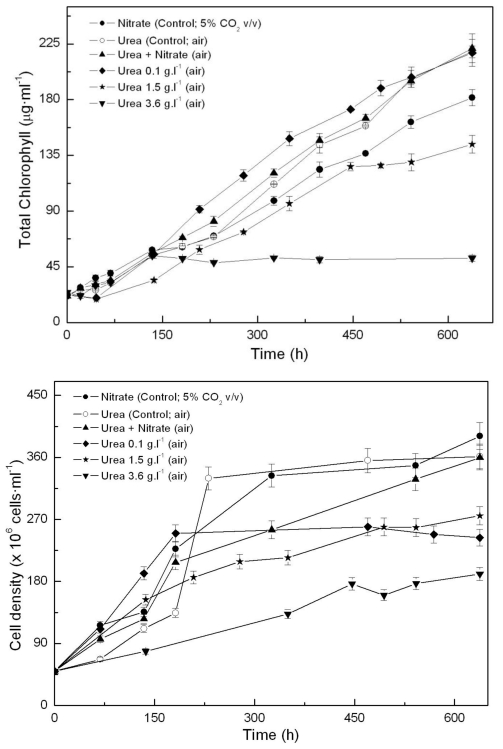
Time-course of chlorophyll (**A**) and cell density (**B**) in *Coccomyxa acidophila* cultures grown on nitrate, urea or nitrate plus urea. Air alone or CO_2_ in air (5% v/v) were used as carbon source, as indicated for each culture within the Figure legend.

One of the main growth conditions assayed was nitrogen source. In previous experiments, we first tested the effect of adding ammonium, nitrite or nitrate to photoautotrophically growing *Coccomyxa acidophila* cells. Growth on ammonium and nitrate resulted in the highest productivities. Unlike common microalgae, nitrite became toxic for *Coccomyxa acidophila*. In the experiments here, we also used urea as a combined source for C and N, with high CO_2_ concentration (5% v.v^−1^) as the main carbon source where indicated. Urea has been widely used instead of high CO_2_ ^−^ for sustaining microalgae growth and is also a cheap N source. As shown in Figure 1, urea promoted enhanced growth of *Coccomyxa acidophila*, both in terms of chlorophyll content (Figure 1A) and cell density (Figure 1B). This resulted in an increased growth rate with respect to control cultures (photoautotrophically grown on nitrate), as shown in Table 1. In addition, culture productivity was higher when the microalga was grown on urea (the highest productivity) and ammonium. Specifically, the highest productivity was reached in cultures grown on 0.67 g·L^·1^ urea (“control; air” in Figure 1). This urea concentration provided cultures with the same molar concentration of nitrogen than the nitrate added to control cultures. More interestingly, the best productivity values obtained from the microalgal growth on urea did not differ from those usually obtained for most of common microalgae (0.2–0.4 g·L^·1^·d^·1^). Unexpectedly, the simultaneous presence of urea and nitrate limited *Coccomyxa acidophila* growth. This will be discussed further.

The previous results were obtained by means of using mixotrophic or photoautotrophic cultures, *i.e.*, either urea or nitrate were added to culture media as N sources while high CO_2_ concentration (5% v/v in air) was supplied as a carbon source (first set of experiments). It has also been discussed that the microalga cells could make use of urea as an additional carbon source [18,19], perhaps being one of the reasons behind the improved microalgal productivity of urea grown cultures. Low CO_2_ solubility at acidic pHs makes carbon uptake more difficult than at pH 7 (standard pH for most common microalgal cultures). Therefore, the supply of additional carbon in a soluble form at low pH (e.g., urea, glucose) could help to increase microalgal productivity. This raises the question of whether addition of glucose as a carbon source to cultures of an acidophila microalga should also increased microalgal productivity. Such a question was investigated by our group in previous research [1] and the answer was “no”. Urea should by far allow maximum productivity in *Coccomyxa acidophila* cultures when used as a carbon source.

**Figure 2:**
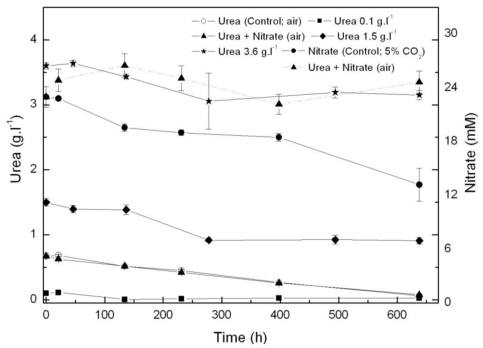
Time course of nitrogen consumption in *Coccomyxa acidophila* cultures grown on nitrate, urea or nitrate plus urea. Air alone or CO_2_ in air (5% v/v) were used as carbon source, as indicated for each culture within the Figure legend. Dotted line with triangles corresponds to time-course of nitrate consumption of cultures incubated with nitrate plus urea.

From the results above, *Coccomyxa acidophila* apparently prefers urea to nitrate as nitrogen source. Therefore, another question we addressed was whether such consumption preference indeed occurred. For this purpose, nitrate and urea consumption were followed in time in photoautotrophic cultures to which nitrate (control culture) or urea and nitrate (with the same molar nitrogen concentration to that used in control cultures, 22.7 mM), were added. Results are shown in Figure 2. If urea and nitrate are added simultaneously, nitrate only started to be consumed at late exponential growth phase while urea was first consumed as the only nitrogen source. A decreasing time-course trend in urea concentration is observed from the beginning of the experiment, whereas the nitrate concentration time-course trend remains stable. Inhibition of nitrate consumption by the presence of urea has been reported to occur in microalgae, though not many references dealing with the subject have been published. Cochland and Harrison [20] reported about 30% inhibition of nitrate consumption by urea in the eukaryotic picoflagellate *Microsomas pusilla*. Following consumption, assimilatory reduction of nitrate also could be inhibited. One of the first classic references was published by Smith and Thompson [21] who observed 70% nitrate reductase inhibition by urea in *Chlorella*, evidencing nitrate assimilatory reduction down regulation to be behind nitrate consumption inhibition by urea.

As already mentioned, simultaneous addition of urea and nitrate as nitrogen sources slightly limits cell growth. Merigout *et al.* [22] evidenced in *Arabidopsis* plants that urea uptake was stimulated by urea but was reduced by the presence of nitrate in the growth medium. Such conclusions from their recent study on physiological and transcriptomic aspects of urea uptake and assimilation are in good concordance with the following observations from our results: (a) urea increased *Coccomyxa acidophila* growth and (b) simultaneous presence of urea and nitrate resulted in a decreased uptake of urea and culture productivity. These observations related to nitrogen uptake regulation in *Coccomyxa acidophila* are for the first time reported in acidophile microalgae and suggest that urea uptake and assimilation patterns in extreme acidophile microalgae (living in fully urea-free environments) and plants are similar. Further experiments in nitrogen assimilatory enzymes and gene expression are currently being developed in our group.

**Figure 3:**
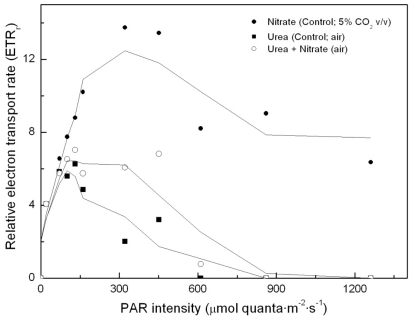
Light-dependent electron transport rates in *Coccomyxa acidophila* cultures grown on nitrate, urea or nitrate plus urea. Air alone or CO_2_ in air (5% v/v) were used as carbon source, as indicated for each culture within the Figure legend.

To determine whether simultaneous addition of urea and nitrate to the algal cultures has any impact on photosynthesis, relative electron transport rates were determined in each of the cultures (namely, control –nitrate–; urea; urea and nitrate, as nitrogen sources, respectively). Results are shown in Figure 3. Surprisingly, there was no nitrogen source-dependent impact on PS2 and on photosynthetic energy production chain, if the light intensity remained approximately below 150 μE·m^−2^·s^−1^. However, in urea grown cultures incubated under higher light intensities, photosynthetic energy production is shown to be clearly limited, up to the point that the electron transport chain becomes inhibited. This dramatically influences carbon assimilation and culture productivity.

According to our results, urea appears to be a suitable nitrogen source for *Coccomyxa acidophila* growth at relatively low light intensity; however, it has a dramatic impact on the photosynthetic energy production chain when exposed to high light intensity, which has never been reported for any other microalga. This is currently under study in our laboratories.

In addition, physiological responses of acidophile microalgae to urea and nitrate uptake processes anyhow differ, according to the observed pH changes in the culture media which tend to increase if nitrate (2.3 g·L^·1^) is the only added nitrogen source and to decrease if urea (0.67 g·L^·1^) is used (data not shown). So far, we have no evidence for antyport/symport mechanism details that help to elucidate the different physiological behavior.

### 2.2. Carotenoid accumulation and xanthophylls cycle activity in urea grown *Coccomyxa acidophila* cells

**Figure 4:**
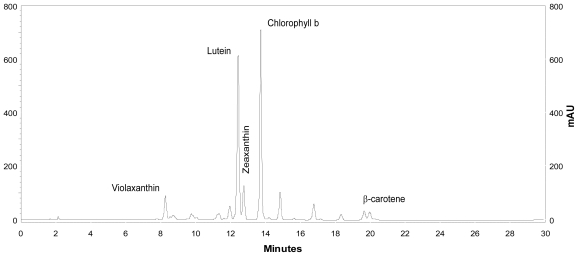
HPLC chromatogram showing the main carotenoids of *Coccomyxa acidophila*. AU: arbitrary units.

**Figure 5:**
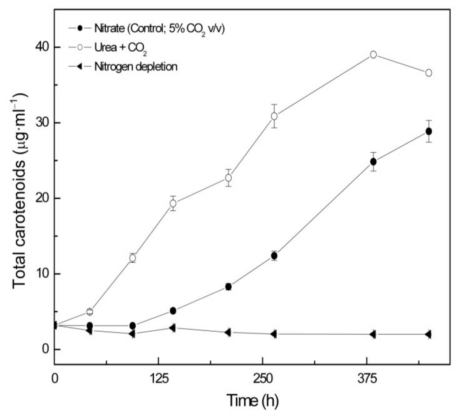
Total carotenoid content in *Coccomyxa acidophila* cultures grown on different nitrogen sources or under nitrogen starvation. Air alone or CO_2_ in air (5% v/v) were used as carbon source, as indicated for each culture within the Figure legend.

**Figure 6:**
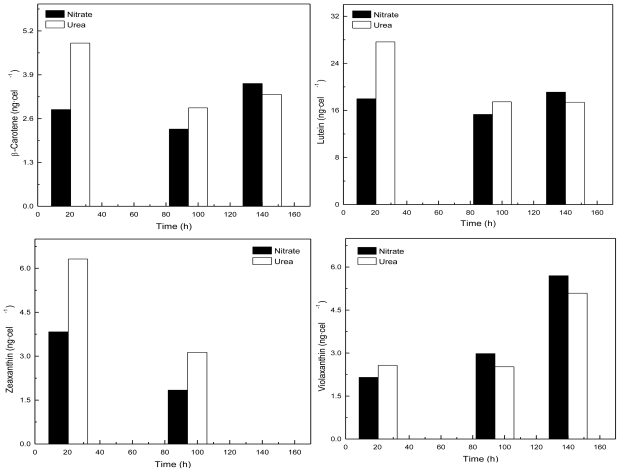
Time-course of the cell content of the indicated specific carotenoids in *Coccomyxa acidophila* cultures incubated in either nitrate or urea.

*Coccomyxa acidophila* accumulates commercial value carotenoids including lutein, β-carotene and zeaxanthin (Figure 4). Besides assessment of the best nitrogen sources for biomass production, carotenoid accumulation in urea and nitrate grown cultures was also studied (Figure 5). According to the best growth conditions inferred from Figure 1, for this experiment, the carotenoid content was followed in urea grown cultures (fluidized with air) and in nitrate grown cultures (fluidized with air supplemented with 5% v/v CO_2_). In addition, carotenoid content was also followed in nitrogen-deprived cultures, as nitrogen depletion is a very well known carotenogenic condition for many microalgae species. In good agreement with the enhanced cell growth in urea grown cultures, total carotenoid content in the reactor also increased much more rapidly in urea grown cultures than in control cultures. Consequently, the carotenoid content of urea grown cultures (μg·mL^·1^) became about two-fold that of the nitrate grown cultures (control cultures), until late exponential growth phase (Figure 5). This could be due to the increased biomass production in urea grown batch cultures, therefore higher carotenoid content in the reactor is not necessarily a consequence of faster carotenoid biosynthesis. However, simple calculations of the content of specific carotenoids per cell revealed a prompt carotenoid biosynthesis enhancement (namely β-carotene, lutein, zeaxanthin) in urea grown *Coccomyxa acidophila* cells, as can be inferred from the carotenoid cell content data in Figure 6; lutein by far being the most abundant carotenoid. This means that urea clearly promotes increases in lutein and β-carotene cell content, at least up to late exponential growth phase, where lack of nutrients, shading effect and stress factors change the observed trend. Besides, it can be observed that cell content of violaxanthin inversely correlates with cell content of zeaxanthin over the time course. This is the first evidence of an active xanthophylls cycle in *Coccomyxa acidophila* that converts violaxanthin into zeaxanthin by means of violaxanthin de-epoxidase activity. Interestingly, nitrogen starvation did not promote carotenoid accumulation in *Coccomyxa acidophila* cultures, unlike other common microalgae including *Dunaliella*, *Haematococcus* and many others [23,24].

Fernández-Sevilla *et al.* [25] recently reviewed the biotechnological production of lutein. The paper includes an updated list of lutein production experiments performed on different scales using microalgae species. Table 2 shows the most relevant data of lutein productivity by microalgae and reactor type used for the production processes. Considering intracellular lutein cell content of each one of the promising species and lutein productivity in photobioreactors, *Scenedesmus almeriensis* [26], *Muriellopsis sp.* [27] and *Chlorella protothecoides* [28] emerge so far as the most efficient strains for the biotechnological production of lutein from microalgae. When incubated under standard culture conditions, *Coccomyxa acidophila* onubensis accumulates up to 6.1 mg·g^·1^ dry weight, which is within the upper range of lutein concentrations accumulated by the above mentioned microalgae. We are now running continuous cultures of *C. acidophila* in tubular laboratory photobioreactors in order to obtain lutein productivity data in long-term (weeks) production processes. Compared to continuous cultivation of other lutein producing species, *C. acidophila* has the practical advantage of growing well in an extremely selective culture medium at very low pH which preserves cultures from microbial contamination.

## 3. Conclusions

The main conclusions of this manuscript are: (1) Mixotrophic cultivation on urea efficiently enhances growth and productivity of *C. acidophile*; signaling strategies towards suitable conditions definition for feasible outdoor production; (2) Urea clearly led to the fastest cell growth; (3) Maximal lutein accumulation was found to occur in urea supplemented culture medium; (4) In addition, algal growth in a shaded culture revealed the first evidence for an active xanthophylls cycle operative in acidophile microalgae.

## 4. Experimental Section

### 4.1. Microorganism and cultivation conditions

*Coccomyxa acidophila,* the algal material used in this work, was isolated from the acidic water of the Tinto River’s mining area, in Huelva (Spain).

Initially, an axenic culture of the microalga was obtained by streaking it on basal agar medium at pH 2.5. After that, isolated colonies were transferred from the solid medium to a liquid medium modified by Silverman and Lundgren [29]. *Coccomyxa acidophila* mother cultures were maintained by periodic transfers in sterile medium adjusted to pH 2.5 with concentrated H_2_SO_4_. Unless otherwise indicated, standard cultivation conditions were batch cultures grown at 25 ºC into 1 L-Roux flasks, bubbled with air containing 5% (v/v) CO_2_ and continuously illuminated with fluorescent lamps (Philips TLD, 30 W, 150 μE·m^−2^·s^−1^ at the surface of the flasks). In those cases where CO_2_ was not supplied to the cultures, it was necessary to put a carbon dioxide trap with KOH 5 M buffer for removing it from the air mix. Every day, pH was controlled and adjusted at 2.5 ± 0.1 by adding diluted HCl or NaOH.

The irradiance was measured with a quantum/photometer Licor (mod. LI–250A).

### 4.2. Dry weight measurements

Before filtering culture samples, filters of cellulose acetate with a 0.45 μm pore size, from Sartorius (Goettingen, Germany), were washed with distilled water and dried at 80 ºC in an oven for 24 h. After that, these were weighted and used to separate cells from the medium. Five milliliter culture samples were taken, vigorously homogenated, and filtered by means of a vacuum pump. Filters containing cells were dried and kept in an oven for 24 h, after which they were weighed.

### 4.3. Measurements of fluorescence

Optimal chlorophyll fluorescence yield measurements (Fm/Fv) were performed with a pulse amplitude modulated fluorometer (Teaching-PAM from WALZ, Effeltrich, Germany). In order to make sure that there is no reduction of the PSII primary electron acceptor Q_A_ and, therefore (consequently), all PSII reaction centers are open, cultures samples of 1 mL were previously adapted to dark conditions for 15 min [30]. After that period, a short saturating pulse of light (SP) was triggered. When necessary (e.g., low chlorophyll concentrations), the PAM modulated light (ML) had to be adjusted to higher values to obtain readings in the proper range.

### 4.4. Oxygen evolution

In addition of fluorescence measurements, the biological activity used to check cell viability was photosynthetic activity. For these determinations, 1 mL samples of *Coccomyxa acidophila* cultures were placed into a Clark-type electrode (Hansatech, U.K.) to measure O_2_-evolution. Measurements were made at 25 ºC in the dark (endogenous respiration) or under saturating white light (1500 μE·m^−2^·s^−1^).

### 4.5. Analytical determinations

Total chlorophyll and carotenoid pigments were determined spectrophotometrically after centrifuging tubes containing samples for 6 min at 13000 rpm, heating them for 1 min, and extracting cell pellets with pure methanol. Sonication by ultrasound was also applied when necessary. After that, samples were spun down again for 5 min at 5000 rpm to eliminate cellular wastes. Calculations were done using equations according to [31].

For specific carotenoid analysis and quantification, separation was performed by liquid chromatography (HPLC; Merck Hitachi) using a RP-18 column with a flow rate of 1 mL/min. The applied gradient was the following (solvent A; ethyl acetate and solvent B; acetonitrile/agua, 9:1 v/v): 0–16 min, 0–60% solvent A; 16–30 min, 60% A; 30 – 35 min, 100% A. In order to quantify, pigment standards supplied by DHI-Water and Environment (Denmark) were injected.

Nitrate was determined following the method described by Cawse *et al.* [32]. Urea was determined according to the method from Wilcox [33].

### 4.6. Statistics

Unless otherwise indicated, all data included in figures and tables represent the average of triplicates.

### 4.7. Cell counting

Cellular density was determined by microscopy using an Olympus CX41 in a Neubauer chamber.

## Figures and Tables

**Table 1:** Growth rates and productivity of *Coccomyxa acidophila* grown on different N-sources.

Nitrogen source	Growth rate (d^·1^)	Maximum productivity (g·L^·1^·d^·1^)	Maximum cellular carotenoids content (pg·cell^·1^)
Nitrate	0.27	0.23	0.084
Nitrite	0.02	0.13	Non detectable
Ammonium	0.31	0.24	0.055
Urea	0.34	0.25	0.104

**Table 2:** Lutein productivity of lutein-enriched microalgae.

Microalga	Lutein (mg·g^−1^)	Lutein productivity (mg·L^·1^·d^·1^)	Cultivation system
*Scenedesmus almeriensis*	5.5 4.5	4.9 mg·L^·2^·d^·1^ 290 mg·m^·2^·d^·1^	Laboratory, continuous culture, 2 L
*Muriellopsis sp*	5.5 4.3	1.4 mg·L^·2^·d^·1^ 7.2 mg·L^·2^·d^·1^	Laboratory, batch, 0.2 L Outdoor, tubular systems, 55 L
*Chlorella protothecoides*	4.6	10 mg·L^·2^·d^·1^	Laboratory, batch, heterotrophic, 16 L
*Coccomyxa acidophila*	6.1	2.0 mg·L^·2^·d^·1^	Laboratory, batch, 2 L
